# Tachycardia As Therapy: Control of an Electrical Storm Through Mechanism-Directed Treatment After Refractory Ventricular Arrhythmias

**DOI:** 10.7759/cureus.114290

**Published:** 2026-08-10

**Authors:** Miguel Santos, Ana Albuquerque, Gonçalo Dias, Fernando Henriques, Odete Gomes

**Affiliations:** 1 Intensive Care Unit, Centro Hospitalar de Leiria, Leiria, PRT

**Keywords:** cardiac electrical storm, electrical storm, idiopathic ventricular fibrillation, isoproterenol, quinidine, refractory ventricular arrhythmias, short-coupled premature ventricular complexes

## Abstract

Electrical storm (ES) is a life-threatening clinical syndrome defined by recurrent episodes of ventricular tachycardia (VT) or ventricular fibrillation (VF) within a short time frame. Although contemporary management relies on guideline-directed therapies, including antiarrhythmic drugs, deep sedation, sympathetic modulation, and catheter ablation in selected refractory cases, some patients remain unstable and require individualized treatment guided by the suspected electrophysiological substrate.

We report the case of a 44-year-old previously healthy woman who presented with out-of-hospital cardiac arrest due to VF. Following successful resuscitation, she developed recurrent polymorphic VT apparently initiated by short-coupled premature ventricular complexes (PVCs). Despite implementation of several conventional and guideline-supported strategies for ES, ventricular arrhythmias continued to recur.

The clinical pattern and electrocardiographic/telemetry recordings were considered compatible with a short-coupled ectopy-triggered mechanism, raising suspicion for a Purkinje-mediated substrate. Based on this hypothesis, a therapeutic strategy aimed at increasing the basal heart rate was adopted using continuous isoproterenol infusion. Following isoproterenol titration to achieve sinus tachycardia of approximately 120 beats per minute, no further VT was documented. Quinidine therapy was subsequently introduced, allowing withdrawal of isoproterenol with sustained arrhythmia suppression.

This case illustrates how careful interpretation of arrhythmia patterns may guide therapeutic decisions in refractory ES and highlights the potential role of heart rate acceleration in selected patients with suspected short-coupled PVC-triggered ventricular arrhythmias.

## Introduction

Electrical storm (ES) is commonly defined as the occurrence of three or more sustained episodes of ventricular tachycardia (VT) or ventricular fibrillation (VF) within 24 hours, requiring intervention [1-3]. This condition is associated with high morbidity and mortality and represents one of the most challenging scenarios in acute cardiac electrophysiology [1-3].

The pathophysiology of ES is heterogeneous and includes ischemia, structural cardiomyopathy, autonomic imbalance, electrolyte disturbances, drug effects, and primary electrical disorders [2,3]. The management of ES typically involves correction of reversible triggers, administration of antiarrhythmic drugs, deep sedation when appropriate, sympathetic modulation with beta-blockers or stellate ganglion blockade, and consideration of urgent catheter ablation or mechanical circulatory support in selected refractory cases [1-3].

However, the optimal treatment strategy depends on the underlying arrhythmogenic mechanism. Idiopathic VF triggered by short-coupled premature ventricular complexes (PVCs), frequently arising from the Purkinje system, has been increasingly recognized as a distinct clinical entity [4-7]. In selected patients, arrhythmia initiation may be facilitated by pauses or slower basal heart rates, and heart rate acceleration may paradoxically reduce triggering ectopy [5,8].

We report a case of refractory ES in a previously healthy patient in whom arrhythmia control was achieved only after adopting a mechanism-guided heart rate acceleration strategy followed by quinidine therapy.

## Case presentation

A previously healthy 44-year-old woman, with no known medical history and no regular medication, suddenly collapsed while working at her cafe. A co-worker who witnessed the event reported that the patient had complained of a sudden headache immediately before losing consciousness. Bystander cardiopulmonary resuscitation was initiated, and the physician-staffed prehospital emergency medical team arrived within two minutes in a medical emergency and resuscitation vehicle. VF was documented as the initial rhythm, and advanced life support was performed in accordance with the European Resuscitation Council Guidelines 2021 [9].

Return of spontaneous circulation was achieved after approximately 20 minutes of advanced life support, during which she received nine defibrillation shocks, 6 mg of adrenaline, and a total of 450 mg of amiodarone (300 mg followed by 150 mg). On arrival of the physician-staffed emergency team, the patient was in return of spontaneous circulation but had gasping respirations and bradycardia. She was sedated with propofol, received rocuronium, and underwent endotracheal intubation without complications. During transport, she suffered a new episode of cardiac arrest with VF as the initial rhythm, requiring one additional defibrillation shock and adrenaline administration, with subsequent return of spontaneous circulation.

On arrival at the emergency department, the patient remained intubated and mechanically ventilated. She was hemodynamically stable, with blood pressure of 105/70 mmHg and sinus rhythm at 80 beats per minute. Initial arterial blood gas analysis showed metabolic acidosis with hyperlactatemia (Table 1). A repeat arterial blood gas analysis under an FiO2 of 60% showed persistent mild acidemia with partial lactate clearance.

**Table 1:** Arterial blood gas analysis during initial assessment

Parameter	Initial arterial blood gas	Repeat arterial blood gas (FiO₂ 60%)	Reference range
pH	7.29	7.29	7.35-7.45
PaCO₂	37 mmHg	44 mmHg	35-45 mmHg
PaO₂	106 mmHg	143 mmHg	80-100 mmHg
HCO₃⁻	17.6 mmol/L	21.2 mmol/L	22-26 mmol/L
Base excess	-8.8 mmol/L	-5.4 mmol/L	-4 to +2 mmol/L
Lactate	8.4 mmol/L	5.9 mmol/L	<2.0 mmol/L
Sodium	138 mmol/L	136 mmol/L	136-146 mmol/L
Potassium	3.2 mmol/L	3.4 mmol/L	3.5-5.1 mmol/L
Chloride	103 mmol/L	107 mmol/L	101-109 mmol/L
Ionized calcium	1.11 mmol/L	1.24 mmol/L	1.15-1.33 mmol/L
Hemoglobin	12.8 g/dL	11.4 g/dL	11.5-16.0 g/dL

**Figure 1:**
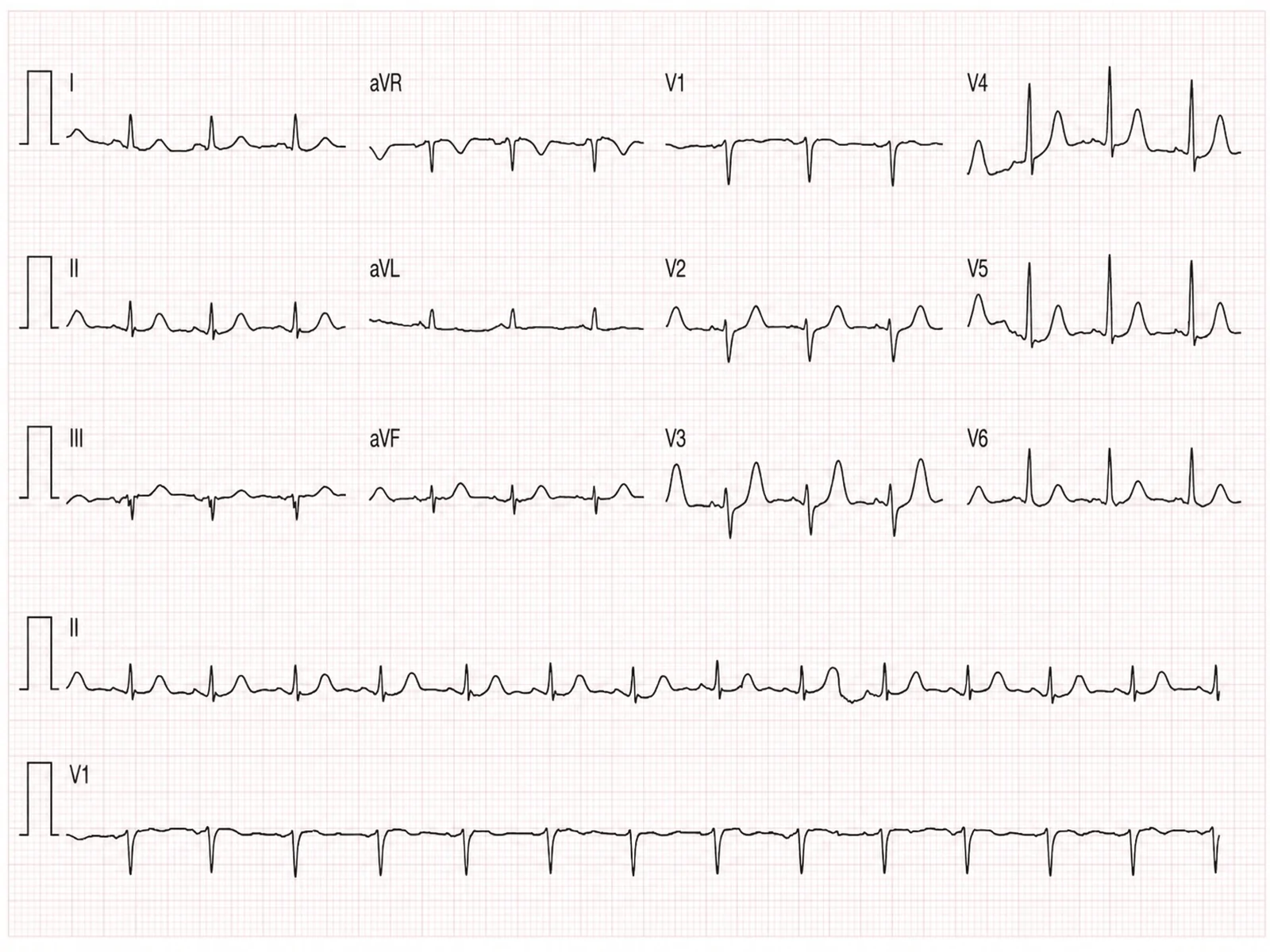
Admission 12-lead electrocardiogram showing sinus rhythm at 83 beats per minute, mild ST-segment depression in V2-V4 (<1 mm), and no ST-segment elevation in standard leads

The admission electrocardiogram showed sinus rhythm at 83 beats per minute, with mild ST-segment depression in leads V2-V4 (<1 mm), without ST-segment elevation in the standard or posterior leads (Figure 1).

A repeat electrocardiogram performed approximately one hour later showed no significant dynamic changes.

**Figure 2:**
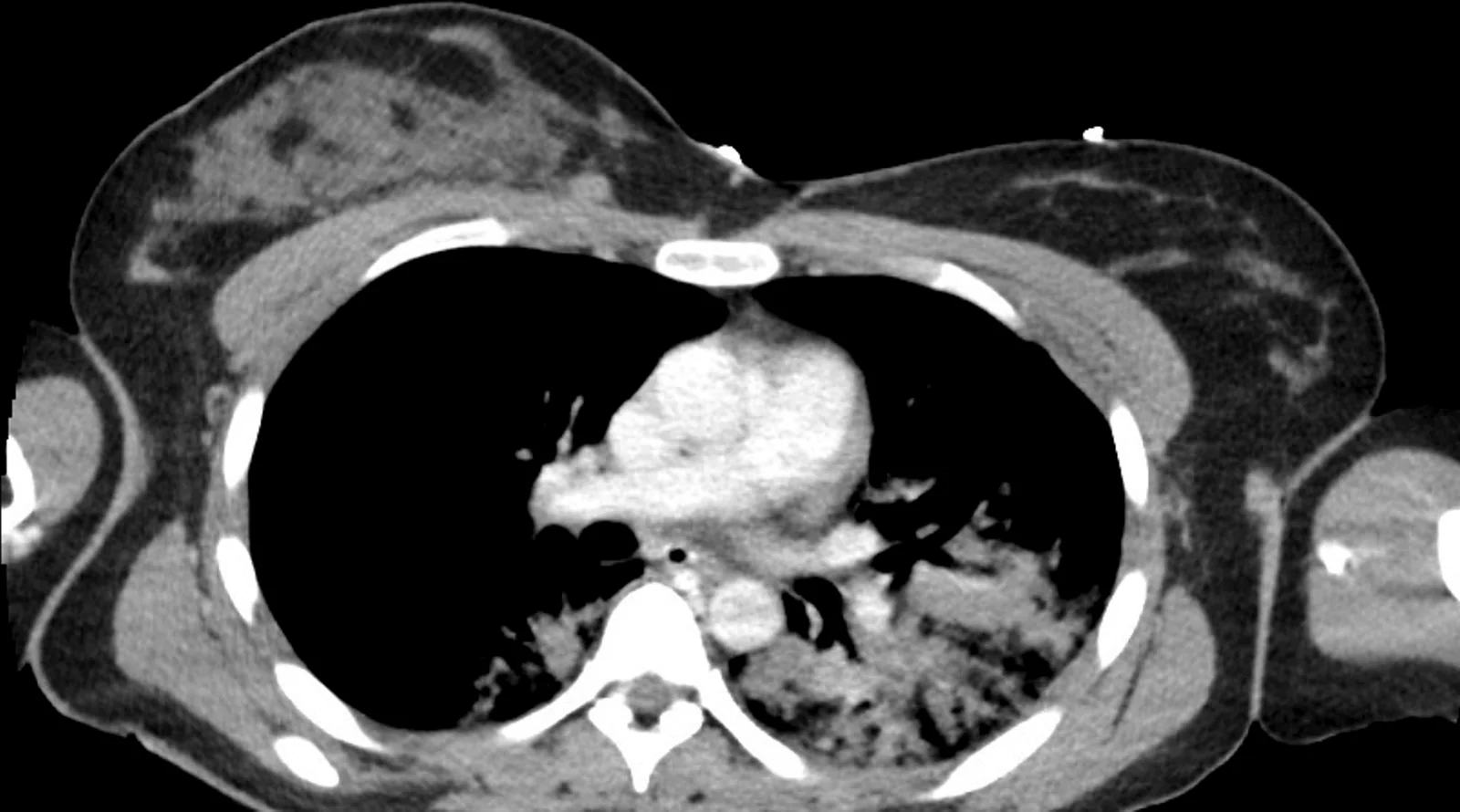
Chest CT pulmonary angiography Representative axial image showing bilateral dependent pulmonary opacities, more pronounced in the left lower lobe. No pulmonary arterial filling defect was identified on the complete examination.

**Figure 3:**
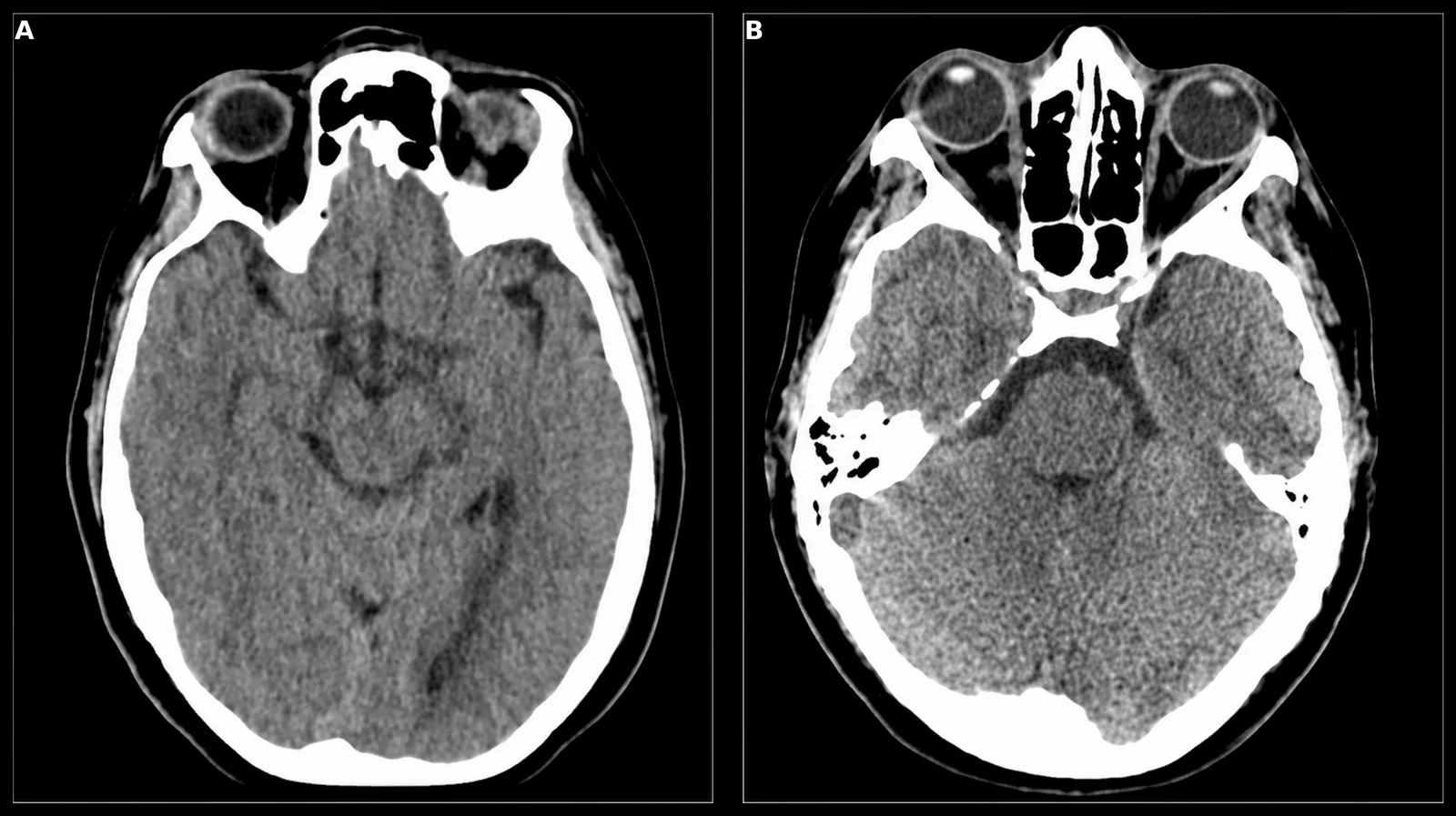
Serial non-contrast brain CT scans (A) Initial examination showing no acute intracranial lesion; (B) Follow-up examination performed 48 hours after admission showing no focal brain parenchymal density abnormality or significant interval change

Point-of-care echocardiography revealed preserved biventricular systolic function, with no evidence of pericardial tamponade and no echocardiographic signs suggestive of pulmonary embolism. Chest CT with pulmonary angiography excluded pulmonary embolism but demonstrated bilateral pulmonary parenchymal opacities, more pronounced in the left lower lobe, likely related to post-resuscitation changes or aspiration (Figure 2). CT of the brain showed no acute intracranial lesions (Figure 3A).

Initial laboratory testing revealed leukocytosis with neutrophilia, markedly elevated cardiac biomarkers, elevated liver enzymes, markedly elevated D-dimer, and preserved renal function (Table 2). Electrolyte abnormalities were limited to mild hypokalemia. Blood alcohol testing and toxicology screening were negative.

**Table 2:** Initial laboratory findings on admission INR: International normalized ratio; ALT: Alanine aminotransferase; AST: Aspartate aminotransferase; LDH: Lactate dehydrogenase; CK: Creatine kinase; TSH: Thyroid-stimulating hormone; T4: Thyroxine

Parameter	Result	Reference range
White blood cell count	19.4 ×10³/µL	4.0-10.0 ×10³/µL
Absolute neutrophil count	16.8 ×10³/µL (86.6%)	1.8-8.0 ×10³/µL
Hemoglobin	13.0 g/dL	11.5-16.0 g/dL
Platelet count	232 ×10³/µL	150-500 ×10³/µL
INR	1.06	0.50-3.00
Activated partial thromboplastin time ratio	1.03	0.80-1.20
D-dimer	31,875 ng/mL	<500 ng/mL
Urea	32 mg/dL	17-43 mg/dL
Creatinine	1.03 mg/dL	0.51-0.95 mg/dL
Sodium	140 mmol/L	136-146 mmol/L
Potassium	3.5 mmol/L	3.5-5.1 mmol/L
Magnesium	1.21 mmol/L	0.77-1.03 mmol/L
Phosphate	2.00 mmol/L	0.81-1.45 mmol/L
Albumin	36 g/L	35-52 g/L
Total bilirubin	9.2 µmol/L	5.0-21.0 µmol/L
ALT	264 U/L	3-34 U/L
AST	271 U/L	15-35 U/L
LDH	556 U/L	100-247 U/L
CK	561 U/L	10-145 U/L
CK-MB	107.0 U/L	5.0-24.0 U/L
Alkaline phosphatase	88 U/L	30-120 U/L
C-reactive protein	0.4 mg/L	≤5.0 mg/L
Procalcitonin	0.12 ng/mL	<0.50 ng/mL
High-sensitivity troponin I	2675.8 pg/mL	<11.6 pg/mL
TSH	10.37 µIU/mL	0.34-5.60 µIU/mL
Free T4	10.6 pmol/L	7.9-14.4 pmol/L
Blood alcohol concentration	<0.1 g/L	---
Toxicology screening	Negative	Negative

The patient was admitted to the ICU for post-cardiac arrest care and neuroprotective management. Deep sedation was maintained with propofol and fentanyl. Neuroprotective measures included maintenance of normothermia, normocapnia, normoxia, and adequate glycemic control. A follow-up brain CT scan performed 48 hours after admission showed no focal brain parenchymal density abnormalities, preserved ventricular and cisternal spaces, patent cortical sulci, no extra-axial fluid or hemorrhagic collections, and no midline shift (Figure 3B). These findings were globally unchanged compared with the initial brain CT scan.

During the first three days of ICU admission, no sustained ventricular arrhythmias were documented. On day 4 of hospitalization, after short self-limited runs of VT, oral beta-blocker therapy with bisoprolol 5 mg was introduced. A few hours later, the patient developed the first in-hospital cardiac arrest due to polymorphic VT.

**Figure 4:**
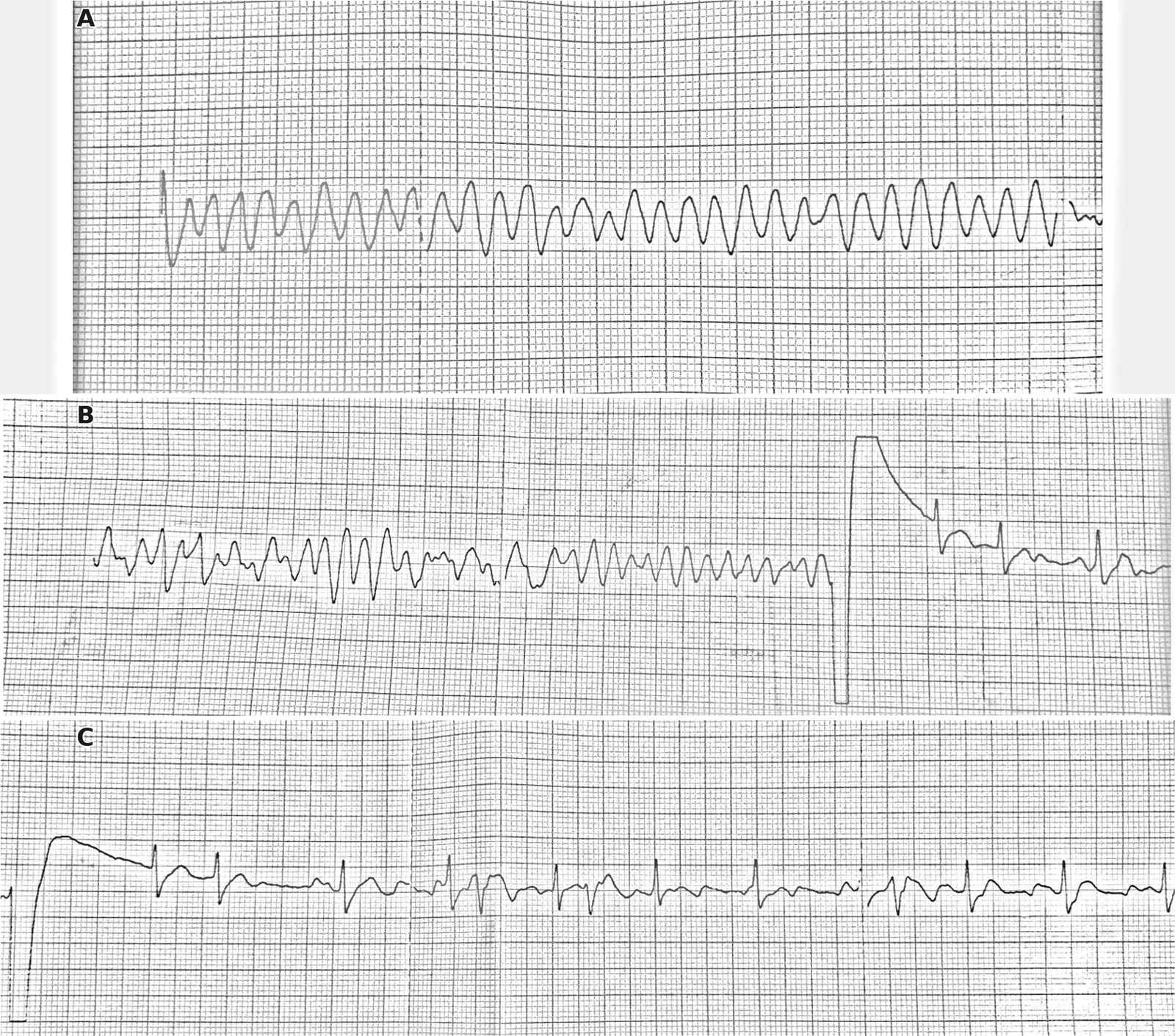
Representative telemetry strips during ES (A) Polymorphic VT immediately before external defibrillation; (B) Longer strip showing polymorphic VT followed by external shock and transition to an organized post-shock rhythm; (C) Post-shock organized rhythm with frequent ventricular ectopy ES: Electrical storm; VT: Ventricular tachycardia

On days 4 and 5 of hospitalization, the patient developed recurrent episodes of polymorphic VT, resulting in three additional cardiac arrests in the ICU. These episodes required immediate resuscitation and defibrillation. At least four defibrillation shocks were delivered during cardiac arrest episodes; additional shocks were also required for episodes of VT with preserved pulse, although the exact number could not be reliably determined retrospectively. Representative telemetry strips are shown in Figure 4.

**Figure 5:**
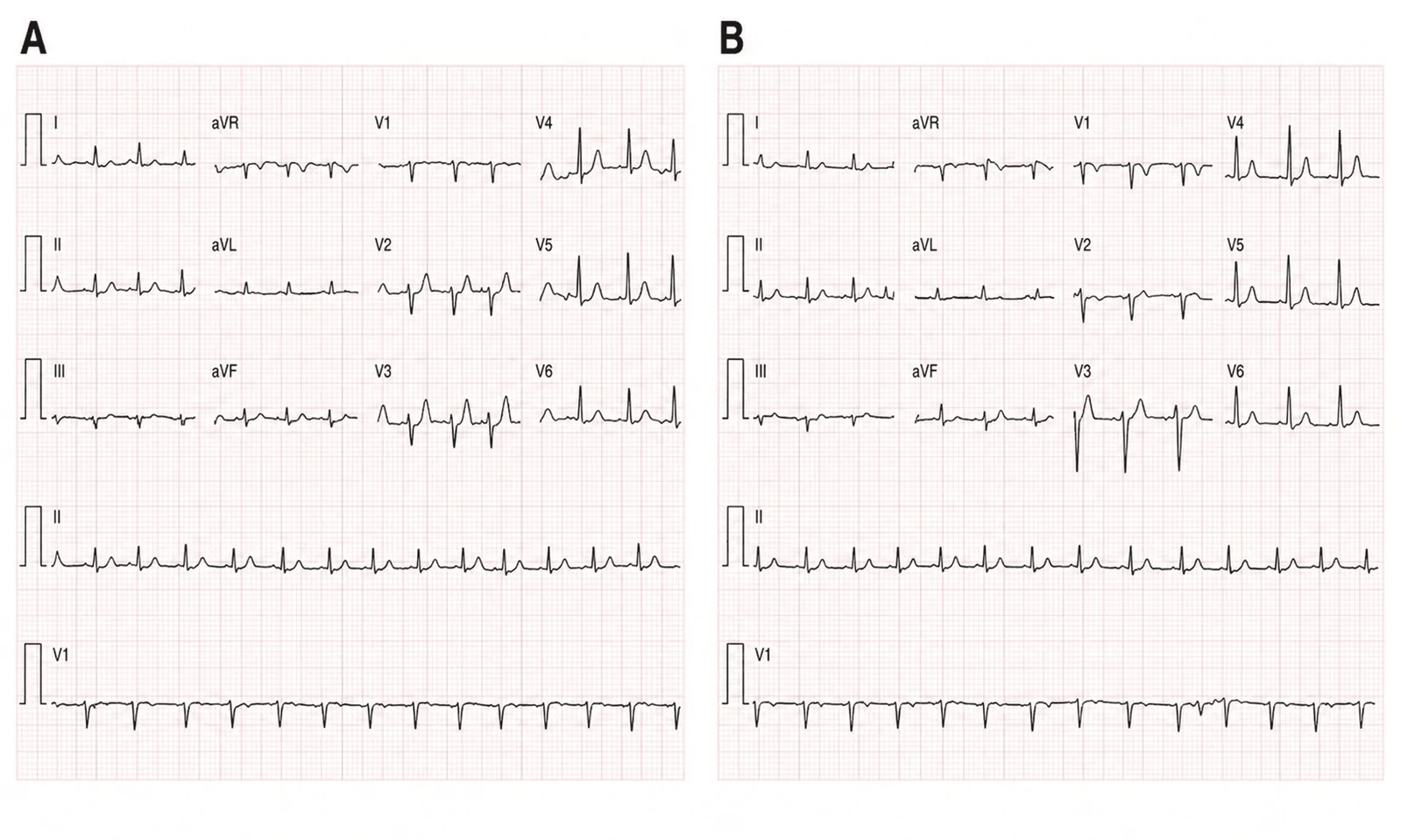
Representative electrocardiograms obtained during arrhythmic recurrence, showing sinus rhythm with frequent ventricular ectopy and short runs of ventricular arrhythmia, compatible with a short-coupled ectopy-triggered mechanism

At this stage, the patient had refractory ES despite electrolyte optimization, beta-blocker therapy, intravenous amiodarone, lidocaine, and left stellate ganglion block. First, the case was urgently discussed with a tertiary cardiac center for consideration of emergent electrophysiological study and catheter ablation. In parallel, a multidisciplinary discussion involving the intensive care and cardiology teams at the referring hospital was undertaken. Review of the electrocardiographic and telemetry recordings (Figures 4, 5) suggested that the recurrent episodes of polymorphic VT were apparently initiated by short-coupled PVCs, raising suspicion for a Purkinje-mediated mechanism.

Following the onset of recurrent ventricular arrhythmias, electrolyte optimization was aggressively pursued. Intravenous magnesium was administered on multiple occasions, and serum magnesium and potassium were maintained within target ranges throughout hospitalization, with magnesium levels between 0.77 and 1.03 mmol/L and potassium levels between 4.5 and 5.0 mmol/L. Intravenous amiodarone was administered as a 300 mg bolus followed by continuous infusion at 900 mg over 24 hours. A single 50 mg intravenous bolus of lidocaine was also given. Despite these measures, recurrent ventricular arrhythmias persisted.

Given the refractory nature of the ES, a left stellate ganglion block was performed as a single ultrasound-guided injection using 8 mL of ropivacaine 0.375%. However, this intervention did not achieve sustained arrhythmia control.

Based on the suspected short-coupled ectopy-triggered mechanism, continuous isoproterenol infusion was initiated at 0.5 μg/min and progressively titrated to 5.0 μg/min, targeting a heart rate of approximately 110-120 beats per minute. No further VT was documented after sustained sinus tachycardia of approximately 120 beats per minute was achieved. Quinidine was subsequently initiated at 200 mg every eight hours, with the first dose administered before transfer to the tertiary cardiac center.

**Figure 6:**
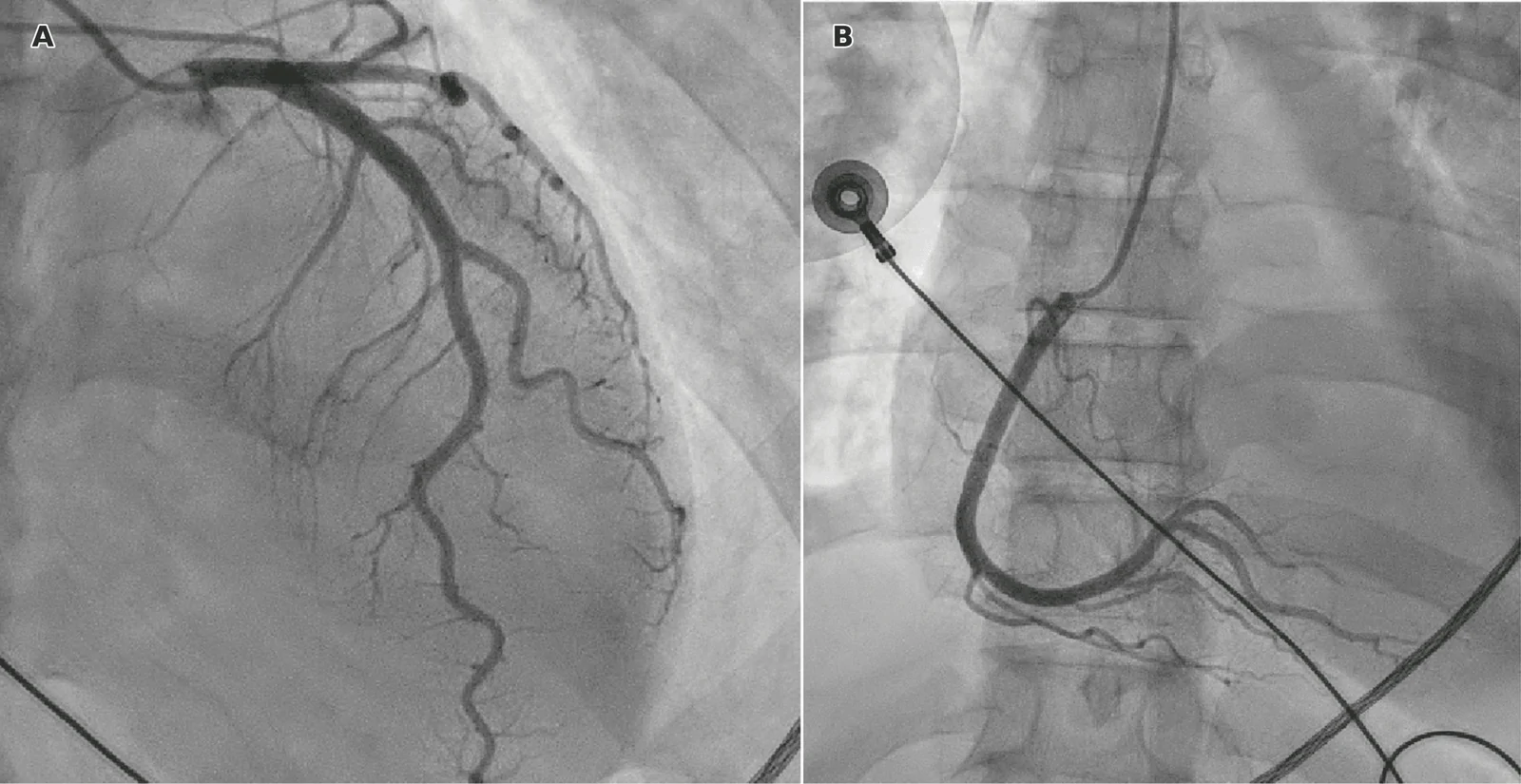
Invasive coronary angiography (A) Left coronary artery; (B) Right coronary artery No significant epicardial coronary artery disease was identified.

Given the absence of ST-segment elevation in both standard and posterior leads, the absence of significant dynamic electrocardiographic changes, and the lack of regional wall motion abnormalities on echocardiography, an acute coronary event was not initially considered the most likely cause of the cardiac arrest. However, as part of the pre-transfer assessment requested by the tertiary center before possible urgent ablation, coronary angiography was performed to exclude an acute coronary substrate. No epicardial coronary artery disease was identified (Figure 6). The patient was transferred immediately thereafter while receiving continuous isoproterenol infusion.

During her stay at the tertiary center, no further ventricular arrhythmias occurred under quinidine therapy, allowing isoproterenol to be discontinued. Although urgent electrophysiological study and catheter ablation had been the reason for transfer, the procedure was deferred because arrhythmia suppression had been achieved and the neurological prognosis remained uncertain at that stage. The patient was subsequently transferred back to the referring hospital while still sedated, intubated, and mechanically ventilated.

Her ICU course was complicated by ventilator-associated pneumonia, with isolation of *Enterobacter cloacae* complex and *Acinetobacter pittii* from tracheal aspirates, which was successfully treated with targeted antibiotic therapy.

Sedation and analgesia were discontinued during the first two days after return from the tertiary cardiac center, and the patient was successfully extubated. She initially presented with a fluctuating level of consciousness, including intermittent periods of confusion and inappropriate or incoherent speech.

Given the presentation with idiopathic VF and ES, a subcutaneous implantable cardioverter-defibrillator was implanted for secondary prevention on hospital day 29, eight days after cardiac MRI. The procedure was completed without complications.

At hospital discharge, the patient was able to transfer out of bed with assistance but still required help with walking and personal hygiene. She was discharged to a rehabilitation facility on hospital day 37, with scheduled cardiology and neurology follow-up.

During subsequent follow-up, repeated device interrogations showed no delivered shocks and no appropriate or inappropriate implantable cardioverter-defibrillator therapies. No further VT recurrence was documented under quinidine therapy. In the absence of recurrent arrhythmias or sufficient spontaneous ectopy to guide mapping, electrophysiological study and catheter ablation were not pursued, even after complete neurological recovery. The patient continued to recover neurologically, eventually regained full autonomy in activities of daily living, and resumed her professional activity.

## Discussion

This case highlights the importance of considering specific arrhythmogenic mechanisms when managing ES, particularly in patients without structural heart disease [1-3].

Idiopathic VF triggered by short-coupled premature ventricular complexes has been increasingly described over the past two decades [5,6]. In many of these cases, the triggering ectopic beats originate from the Purkinje network and can initiate polymorphic VT or VF [4,6,7,10].

The diagnosis of idiopathic VF in this patient was supported by the absence of epicardial coronary artery disease, preserved ventricular function on echocardiography, absence of myocardial fibrosis or late gadolinium enhancement on cardiac MRI, negative genetic testing using a cardiomyopathy- and arrhythmia-associated gene panel, an unremarkable 24-hour Holter recording, and negative family screening. Nevertheless, the absence of provocative testing with ajmaline or flecainide limits the complete exclusion of concealed sodium-channel-related phenotypes.

The Purkinje system is particularly susceptible to triggered activity and early afterdepolarizations [10,11]. When premature beats occur with very short coupling intervals, they may encounter heterogeneous myocardial refractoriness, creating conditions that favor the initiation of reentrant ventricular arrhythmias [5,11]. In contrast to many arrhythmias associated with structural heart disease, short-coupled ectopy-triggered arrhythmias may, in selected cases, be facilitated by slower heart rates or pauses [4,5]. As a result, increasing the basal heart rate may suppress arrhythmia initiation by reducing opportunities for pause-dependent triggering and modifying dispersion of refractoriness [5,8].

This case also illustrates the difficulty of early therapeutic decision-making in ES. Before the triggering pattern was fully recognized, beta-blocker therapy was introduced in response to recurrent non-sustained VT, in accordance with conventional approaches to ventricular arrhythmia suppression. A causal relationship between beta-blocker administration and subsequent arrhythmic deterioration cannot be established. However, subsequent analysis suggested a pause- or bradycardia-facilitated mechanism, which ultimately supported a shift toward heart rate acceleration rather than further rate-slowing therapy.

In the present case, isoproterenol titration to maintain sinus tachycardia was temporally associated with suppression of ventricular arrhythmias. Although causality cannot be definitively established in a single case, this temporal association supports a clinically meaningful role for heart rate acceleration in this specific context. Heart rate acceleration is not a standard first-line therapy for most cases of ES, but it has been reported to be effective in selected pause-dependent ventricular arrhythmias [6,10].

Quinidine likely played a central role in long-term arrhythmia suppression. This drug blocks several ionic currents, including the transient outward potassium current, thereby reducing heterogeneity of ventricular repolarization [12]. Quinidine has demonstrated benefit in malignant arrhythmia syndromes, including idiopathic VF and Brugada syndrome [12]. In our patient, ventricular arrhythmias did not recur after quinidine initiation, allowing isoproterenol withdrawal.

A relevant limitation of this case is that an electrophysiological study with mapping and catheter ablation was not performed. Ablation of Purkinje triggers may be effective in idiopathic VF when the triggering ectopy is identifiable and mappable [4,6,7]. Importantly, urgent transfer to the tertiary cardiac center was undertaken for possible emergent catheter ablation in the setting of refractory ES. In parallel, the referring intensive care and cardiology teams adopted a mechanism-guided strategy based on the apparent short-coupled premature ventricular complex-triggered pattern. By the time invasive electrophysiological treatment was being considered, arrhythmia suppression had been achieved with heart rate acceleration and quinidine, while the neurological prognosis remained uncertain. Although the patient subsequently recovered neurologically, no further VT occurred and no sufficient spontaneous ectopy was documented to guide mapping. For this reason, invasive electrophysiological study and ablation were not pursued during follow-up.

Another limitation is that the suspected Purkinje-mediated mechanism was inferred from the arrhythmic pattern, the apparent initiation by short-coupled premature ventricular complexes, the absence of structural heart disease, and the response to heart rate acceleration and quinidine. Without invasive mapping, this mechanism cannot be considered definitively proven. The case should therefore be interpreted as a clinically plausible example of mechanism-guided therapy rather than a definitive demonstration of a Purkinje trigger.

An additional limitation is that the precise PVC coupling interval and baseline QTc could not be measured reliably from the available electrocardiographic and telemetry recordings. This limits the quantitative characterization of the initiating ventricular ectopy and the baseline repolarization profile.

More broadly, this case illustrates an important clinical principle: management of ES should not rely solely on protocol-based treatment but should also consider the underlying electrophysiological substrate. In selected refractory cases, careful review of the arrhythmia initiation pattern may identify therapeutic strategies that initially appear counterintuitive but are mechanistically appropriate.

## Conclusions

ES is a life-threatening condition requiring rapid and coordinated management. Although current guidelines provide a framework for treatment, refractory cases may require individualized therapeutic strategies based on the suspected arrhythmogenic mechanism.

In patients with ventricular arrhythmias apparently triggered by short-coupled premature ventricular complexes, a Purkinje-mediated mechanism should be considered, particularly when structural heart disease and acute coronary disease have been excluded. In selected cases, heart rate acceleration may help suppress arrhythmia initiation.

This case highlights the importance of integrating clinical observation, electrocardiographic interpretation, electrophysiological reasoning, and mechanism-directed therapy when treating complex ventricular arrhythmias.
